# Associations of Psychosocial Factors with Multiple Health Behaviors: A Population-Based Study of Middle-Aged Men and Women

**DOI:** 10.3390/ijerph17041239

**Published:** 2020-02-14

**Authors:** Kristin Thomas, Evalill Nilsson, Karin Festin, Pontus Henriksson, Mats Lowén, Marie Löf, Margareta Kristenson

**Affiliations:** 1 Department of Health, Medicine and Caring Sciences, Faculty of Medicine and Health, Linköping University, SE-581 83 Linköping, Sweden; evalill.nilsson@liu.se (E.N.); karin.festin@liu.se (K.F.); pontus.henriksson@liu.se (P.H.); mats.lowen@regionostergotland.se (M.L); margareta.kristenson@liu.se (M.K.); 2 Department of Biosciences and Nutrition, Karolinska Institute, 141 83 Huddinge, Sweden

**Keywords:** multiple health behaviors, psychosocial factors, lifestyle factors, health behavior change

## Abstract

*Background*: The health behaviors smoking, risky alcohol consumption, insufficient physical activity, and poor diet constitute the main contributors to non-communicable diseases. Clustering of risk behaviors is common and increases the risk of these diseases. Despite health benefits, it is difficult to change health behaviors. Psychosocial factors could play a role in health behavior change, with research showing relationships between unfavorable psychosocial factors and health risk behaviors. However, many studies only investigated one or two health behaviors at a time. The present study, therefore, aimed to investigate associations between a broad range of psychosocial factors and multiple health risk behaviors in a general middle-aged population in Sweden. *Methods:* A cross-sectional design was used to investigate a random sample from the general population in Sweden (*n* = 1007, 45–69 years, 50% women). Questionnaire data on health behaviors (smoking, alcohol consumption, physical activity, and fruit/vegetable intake) and psychosocial factors, with both psychological and social resources (social integration, emotional support, perceived control, self-esteem, sense of coherence and trust) and psychological risk factors (cynicism, vital exhaustion, hopelessness and depressiveness), were analyzed. Logistic and ordinal logistic regression were used to analyze associations between psychosocial factors and multiple (0–1, 2 or 3–4) health risk behaviors. *Results:* A total of 50% of the sample had two health risk behaviors and 18% had three health risk behaviors. After adjusting for age, sex, education, employment status, and immigrant status, eight out of 10 psychosocial factors (exceptions: social integration and self-esteem) showed significant odds ratios (ORs) in the expected directions; low levels of psychosocial resources and high levels of psychosocial risk factors were associated with multiple risk behaviors. The strongest associations with multiple risk behaviors were seen for vital exhaustion (adjusted (adj.) OR 1.28; confidence interval (CI) 1.11–1.46), depressiveness (adj. OR 1.32, CI 1.14–1.52), and trust (adj. OR 0.80, CI 0.70–0.91). When controlling for all psychosocial factors in the same model, only the association with trust remained statistically significant (adj. OR 0.89, CI 0.73–1.00, *p* = 0.050). Associations with individual health behaviors were fewer and scattered, with no psychosocial factor being related to all four behaviors. *Conclusions*: Examining associations between a broad range of psychosocial factors and multiple health risk behaviors revealed consistent and significant associations for almost all psychosocial factors. These associations were stronger compared to associations to single health risk behaviors. Our findings support the relevance of considering psychosocial aspects in interventions aimed at health behavior change, especially for people with multiple health risk behaviors.

## 1. Introduction

The role of health behaviors in the development of the major non-communicable diseases, i.e., cardiovascular disease, cancer, respiratory disease, and type II diabetes, is well described. Health behaviors in terms of “the big four” (tobacco use, alcohol intake, physical inactivity, and dietary habits) are substantial contributors to the global burden of disease [1,2] and account for more than 50% of preventable premature deaths globally [3].

Health behaviors were found to cluster within the same individual, and the majority of adults report two or more risk behaviors [4,5,6]. For example, smokers—compared to non- and ex-smokers—are more likely to report physical inactivity, poor diet, and risky alcohol consumption [7]. Clustering in terms of risky alcohol consumption and smoking, as well as clustering of all four risk behaviors, was also noted [8]. In addition, clustering of risk behaviors is more prevalent in low socioeconomic groups (income and education) [6,9].

Engaging in multiple risk behaviors was shown to constitute a risk of disease development that is greater than the summated risk for each of the behaviors, for both general populations [10] and clinical populations [11]. Individuals with four risk behaviors compared with those who report one risk behavior have a two-fold risk of stroke [12] and three times the risk of coronary vascular disease and cancer mortality [13]. In addition, individuals engaging in four risk behaviors have an all-cause mortality risk corresponding to being 12 years older than people with no risk behaviors [13].

The last decade saw an increase in interventions that target several behaviors concurrently [14]. A scoping review of the characteristics of these interventions showed that components such as health education, advice, and skills training were common [14]. However, a meta-review reported limited effectiveness of methods for multiple risk behavior interventions in terms of reducing heart disease risk. Interventions included face-to-face sessions and written materials for health behavior change [15]. Similarly, a recent meta-analysis showed limited support for the examined methods for multiple risk behavior interventions. Indeed, interventions largely involving education and skills training resulted in small improvements in diet, physical activity, and smoking cessation in non-clinical populations [16]. The authors suggest that, even though factors such as information and skills training are important for behavior change, other behavior-influencing factors should also be considered, e.g., social support [16]. Psychosocial factors such as psychological resources and social support could, therefore, be important factors in order to understand health behaviors [17,18]. 

Psychosocial factors are characteristics or facets that influence an individual psychologically and/or socially. Such factors can describe individuals in relation to their social environment and how these affect physical and mental health. Psychosocial factors include protective psychosocial resources and psychological risk factors. Psychosocial resources in the social environment include social network and social support. Central among psychological resources are coping ability or mastery, sense of coherence, and self-esteem. Psychological risk factors include vital exhaustion, depressiveness, hopelessness, and hostility.

Coping can be defined as a positive response outcome expectancy [19]. This expectancy is based on the interaction between an exposure and the response to the same exposure. When the result is negative, the individual stores this experience as negative outcome expectancy and feels “hopeless”. If the individual learns that there is no relationship between his or her responses and the outcome, the individual develops “helplessness” [19]. If an individual perceives a situation as manageable, it promotes feelings of coping and mastery. Many of these expectancies represent learning that occurs early in life and contributes to future learning episodes, and they may influence understanding, motivation, and adherence to lifestyle decisions [19]. The concept of “sense of coherence” covers the ability to define life events as less stressful (comprehensibility), to mobilize resources to deal with encountered stressors (manageability), and experience motivation, desire, and commitment to cope (meaningfulness) [20]. The perception of self-esteem, depicting feelings of self-worth [21], is also a prevalent psychological resource in the literature. Furthermore, Bandura introduced the concept of self-efficacy referring to one’s belief in the ability to perform a specific behavior, e.g., physical activity. Bandura differentiates between self-efficacy and outcome expectations. While the latter refers to the anticipation of results of one’s own action, the former refers to one’s ability of performing a certain action. High perceived self-efficacy enables a person to cope with confidence and high motivation [22]. The coping scale developed by Pearlin [21] aims to capture feelings of mastery, that is, feelings of control over one’s life, i.e., internal control.

Furthermore, theory and empirical data posit that, when external demands are perceived to be larger than available protective resources, it can lead to experiences as negative outcome expectancy, to hopelessness and helplessness, and to the increase of what we define as psychological risk factors. These include negative feelings and emotions (e.g., vital exhaustion and depressiveness) and cognitions (e.g., hopelessness and hostility). The effects of psychosocial factors on the risk of disease were proposed to follow two main pathways, the direct psychoneuroendocrine pathway and the indirect pathway through health behaviors [23,24]. 

Furthermore, the relationship between psychosocial factors and health behaviors was reported in large population-based studies in terms of psychological resources (e.g., sense of coherence [25,26,27,28], mastery and resilience [29,30], self-esteem [31,32,33]) and psychological risk factors (e.g., depressiveness [34,35,36], cynicism [25,37]). However, the majority of previous studies investigated one or two health risk behaviors and only included individual or few psychosocial factors [27]. Considering the increased risk of disease from multiple risk behaviors and the reality that most adults report two or more risk behaviors, it is essential to investigate the determinants of multiple risk behaviors rather than simply studying individual risk behaviors.

The aim of the present study was to investigate the association between a broad range of psychosocial factors and multiple risk behaviors (smoking, alcohol consumption, physical activity, and fruit/vegetable intake) in a middle-aged general population in Sweden. Psychosocial factors included social resources (social integration and emotional support), psychological resources (perceived control, self-esteem, sense of coherence, and trust), and psychological risk factors (cynicism, vital exhaustion, hopelessness, and depressiveness). The hypothesis was that psychological and social resources would be negatively associated with health risk behaviors with the opposite association for psychological risk factors.

## 2. Methods

### 2.1. Study Design, Population, and Procedure

The present study is a part of the Life Conditions, Stress, and Health (LSH) research program, which aims to, prospectively, investigate the causes of socioeconomic status differences in the incidence of coronary heart disease (CHD). Data collection was conducted in collaboration with 10 primary care centers in the County of Östergötland, Sweden from October 2003 to May 2004. 

Data collection included questionnaire data on health behaviors, psychosocial factors, and demographic data. A random sample of participants from the general population aged 45 to 69 (i.e., in an age group where the risk of future CHD starts to significantly increase) was invited to take part [38]. Participants were invited consecutively until a study sample size of *n* = 1007, stratified by five-year age groups and sex. The exclusion criterion was serious medical conditions that would interfere with practical procedures. With a response rate of 62.5%, the final study population included 505 women and 502 men. The sample was representative of the general population in terms of educational attainment, employment rates, and immigrant status at the time of data collection. Table 1 shows demographic characteristics, health behaviors, and socioeconomic status of the study population. Table 2 shows the prevalence of health behaviors and their combinations. The present study is a cross-sectional analysis of these data.

### 2.2. Data

#### 2.2.1. Health Behaviors

Four health behaviors were investigated in the present study. *Smoking* was based on a single item, “Do you smoke?”, with four response options: (1) No, I have never smoked, (2) No, I have quit smoking, (3) Yes, sometimes, but less than one cigarette a day, and (4) Yes, regularly, at least one cigarette a day. Dichotomized response options were used in the data analyses whereby response options 3 and 4 were categorized as current smoker and the others as non-smokers. 

*Intake of fruit and vegetables* was used as a proxy for healthy dietary behaviors. Items investigating intake of fruit and vegetables were retrieved from a 13-item food frequency questionnaire [39] measuring consumption of broccoli, cabbage, carrots, cauliflower, onion, root vegetables, spinach, tomatoes, peas, beans, apples, bananas, oranges, fruit juice (maximum 100 g per day), and berries. For each item, participants reported monthly, weekly, or daily intake. Each response option (e.g., monthly) was converted to a daily frequency. These frequencies were then multiplied by an age- and gender-specific portion size to calculate the summarized daily intake of fruit and vegetables. Finally, the data were dichotomized to insufficient (<500 grams per day) and sufficient intake (>500 grams per day, including a maximum of 100 g of fruit juice) according to the Swedish National Food Agency’s recommended intake [40]. 

*Alcohol intake* was retrieved from three questions regarding consumption of beer, wine, and spirits in terms of standard glasses [39]. Risky alcohol drinking was operationalized as follows: drinking more than nine standard glasses per week for women and more than 14 glasses per week for men, and/or reporting drinking four or more standard glasses for women and five or more glasses for men on a typical day when drinking. One standard glass is equivalent to 12 grams of pure alcohol.

*Physical activity* was assessed using two questions on daily activity: one on physical activity on a daily basis, and one on physical exercise. These were combined into an index with four categories: hardly any physical activity, some physical activity, could be more physically active, and enough physical activity. The three lowest categories were then combined into insufficient level of physical activity [41].

Daily smoking, insufficient fruit and vegetable intake, risky alcohol drinking, and/or insufficient level of physical activity are described as health risk behaviors in the present study.

#### 2.2.2. Psychosocial Factors 

A broad range of validated psychosocial scales were used to measure psychosocial resources and psychological risk factors. These were included in the LSH research program because of their known relationships with incident CHD [42]. Further information on these scales is shown in Table 3 and Table 4. 

#### 2.2.3. Psychological and Social Resources

To investigate factors in the social environment, two parts from the abbreviated version of the Interview Schedule Interaction (ISSI for Social) were used. Availability of social interaction (AVSI, six items) investigated *social integration*, i.e., the availability of social contacts such as friends, work associates, and acquaintances, while availability of attachment (AVAT, six items) investigated *emotional support*, i.e., the availability of close relationships [43]. 

*Perceived control* over health and life was measured using an adapted version of a questionnaire from New Barometer studies [44]. *Self-esteem* was measured using the Rosenberg Self-Esteem Scale [45]. The scale’s items investigate a global dimension of self-worth and self-esteem compared to other people’s competences. *Sense of coherence* (SOC) was measured using Antonovsky’s [46] scale investigating the extent to which life is perceived to be comprehensible, manageable, and meaningful. *Trust* was measured with a single item, “Do you feel that, in general, you can trust people?” [47].

#### 2.2.4. Psychological Risk Factors

*Cynicism* was measured using the Cook–Medley Hostility Scale which investigates participants’ general view of humankind [48]. *Vital exhaustion*, e.g., fatigue, irritability, and depressed affect, was investigated using a short version of the Maastricht Questionnaire [49,50]. Feelings of *hopelessness* were investigated using the Hopelessness Scale, where items aim to capture negative expectancies about oneself and the future [51]. Finally, the Center for Epidemiological Studies Depression Scale was used to investigate *depressiveness* and aimed to capture the frequency of low mood, e.g., feeling lonely, but not identifying clinical depression [52]. 

#### 2.2.5. Demographic Data and Socioeconomic Status

Age, sex, and socioeconomic status in terms of education, employment, and immigrant status were reported via the questionnaire. The level of education was investigated with a question on the highest level of education achieved, and the data were categorized into four groups according to the Swedish formal education system: (1) 6–9 years of formal education, (2) 10–11 years, (3) 12–13 years, and (4) ≥14 years (university); only category 1 is mandatory for all Swedish citizens. Employment status was assessed as being employed, unemployed, or retired, and immigrant status as being born inside or outside of the Nordic countries.

### 2.3. Statistical Data Analysis

Health behaviors, psychosocial factors, and demographic data were described as numbers and percentages, means (standard deviation, SD), or medians (interquartile range). The internal consistency of the psychosocial scales was measured using Cronbach’s alpha, except for trust which only contained one item. Internal consistency ranged from 0.68 (depressiveness) to 0.94 (vital exhaustion), which are all in the acceptable range for use at group level. Spearman correlation coefficient was used for the analyses of the intercorrelations between the psychosocial factors. Health behaviors were dichotomized according to national Swedish surveys and Sweden’s national recommendations were used to examine health behavior measures. For psychosocial scale scores, Z-standardized continuous data were used. For analyses of multiple health risk behaviors, data were divided into 0–1 versus 2 and 3–4 health risk behaviors.

Logistic regression analyses were applied to investigate the relationship between psychosocial factors and each of the four health behaviors, adjusted for sex, age, education, employment, and immigrant status. This was done using one model for each of the 10 psychosocial scales. In the main analysis, ordinal logistic regression analyses were applied to investigate these 10 psychosocial factors against having 0–1 versus 2 and 3–4 risk behaviors, with odds ratios for falling in a higher level of health risk behaviors. The assumption of proportional odds was met. The ordinal logistic regression models were adjusted for sex, age, education, employment, and immigrant status. In a second stage, these models were corrected also for all other psychosocial factors. For the final model, multicollinearity was checked with variance inflation factor (VIF) and tolerance statistics. All the VIF values were below 10 (the highest being 2.8) and the tolerance statistics were all above 0.2 (the highest 0.38). Therefore, we concluded that there was no collinearity in our analyses. Odds ratios (ORs) are presented with 95% confidence intervals (CIs). A two-sided probability value of *p* ≤ 0.05 was considered statistically significant. Multiple-comparison correction was carried out according to false discovery rate (FDR). Analyses were performed in SPSS, release 24. 

### 2.4. Statement of Human Rights 

All procedures performed in the study were in accordance with the ethical standards of the regional research committee and with the 1964 Declaration of Helsinki and its subsequent amendments or comparable ethical standards. The study was approved by the Regional Ethics Review Board of Linköping University, Linköping, Sweden (02–0324).

### 2.5. Informed Consent

Written informed consent was obtained from all individual participants included in the study.

## 3. Results

Table 1 presents demographic characteristics, health behaviors, and socioeconomic status in the study population. The participants were on average 57 (SD 7.2) years old, and half of them were women. About one-third of the study population only attended compulsory school. About 80% reported an insufficient intake of fruit and vegetables, 19% were current smokers, 14% reported a risky level of alcohol consumption, and 80% were insufficiently physically active. A clear majority (71%) of the study population reported two or more health risk behaviors (50% reported two, 18% reported three, and 3% reported four health risk behaviors); 24% reported one health risk behavior, and 5% reported none.

Among those with two or three health risk behaviors (Table 2), insufficient physical activity and insufficient intake of fruit and vegetables were most commonly combined with only one additional health risk behavior. Current smoking and risky levels of alcohol intake were most commonly combined with two additional health risk behaviors. Notably, most smokers (88%) reported three or four risk behaviors.

The results for the psychosocial scales are presented in Table 3. Generally, the scale scores in the study population covered the theoretical range in the scales well. Table 4 presents correlation coefficients between psychosocial factors. With the exception of vital exhaustion and depression (*r* = 0.67) and vital exhaustion and SOC (*r* = 0.6), the correlation coefficients were low to large ranging between 0.13 and 0.53.

Table 5 shows relationships between individual health risk behaviors and psychosocial factors. After adjusting for sex, age, education, employment, and immigrant status, significant relationships were seen for three of the four health behaviors (exception fruit and vegetables) with both psychosocial resources and risk factors in the hypothesized directions (OR < 1 for resources and OR > 1 for risk factors relative to health risk behaviors). As indicated in the table, the relationships remained statistically significant for most of the factors (smoking: four of five factors; alcohol intake: two of four factors; physical activity: four of six factors). 

Table 6 shows results after investigating the relationships between psychosocial factors and multiple health behaviors. After adjusting for age, sex, education, employment, and immigrant status, statistically significant relationships were seen for nine out of 10 psychosocial factors (the exception was self-esteem). After multiple-comparison correction according to false discovery rate (FDR), statistically significant relationships remained for eight out of 10 psychosocial factors (except social integration and self-esteem). The strongest associations were seen for vital exhaustion (adjusted (adj.) OR 1.28; CI 1.11–1.46), depressiveness (adj. OR 1.32, CI 1.14–1.52), and trust (adj. 0.80, CI 0.70–0.91). When controlling for all psychosocial factors in the same model, only the association with trust remained statistically significant (adj. OR 0.89, CI 0.73–1.00, *p* = 0.050).

## 4. Discussion

This study investigated the association between a broad range of psychosocial factors and multiple health behaviors in a population-based sample of Swedish middle-aged men and women. The findings showed that eight out of ten of the psychosocial factors investigated were related to having multiple risk behaviors. The results were all in the expected direction, with low levels of resources and high levels of psychosocial risk factors being related to higher risk for multiple risk behaviors. In particular, associations were more prominent when analyzing multiple risk behaviors compared with analyzing associations with single health risk behaviors.

Our study confirmed that clustering of health risk behaviors is common. Indeed, 50% and 21%, respectively, reported two or more risk behaviors [4,5,6]. Our findings also showed that physical inactivity together with low intake of fruit and vegetables was the most common combination of behaviors. In addition, most smokers (88%) reported three or four risk behaviors. Previous studies showed similar findings, whereby smokers are more likely to report insufficient physical activity, low intake of fruit and vegetables, and high alcohol consumption than non- and ex-smokers [7]. 

Clustering of health risk behaviors has implications for health promotion and practice. Presenting multiple risk behaviors determines the conditions for health behavior change and how any intervention is optimally targeted. To understand this common clustering of health behaviors, it is important to identify factors that underpin different behaviors. Earlier studies on health behaviors categorized behaviors as addictive (smoking and risky drinking of alcohol) and health-enhancing (physical activity and healthy eating) behaviors. The two groups of behaviors encompass different processes of behavior change and maintenance, and may also constitute different driving forces, e.g., refraining from having a cigarette versus actively seeking out healthy foods [53].

Theories of behavior change typically argue that constructs such as motivations, self-regulation, resources (psychological and physical), habits, and environmental cues determine actual behavior. Subsequently, health behavior interventions include techniques that aim to tap into these determinants, e.g., self-monitoring of a behavior, or advice and support in the decision-making process [54]. Our findings emphasize the role of psychosocial factors in health behavior interventions, specifically regarding multiple behaviors. In the emerging field of behavioral cardiology, the interplay between conventional and psychosocial risk factors is emphasized [55]. One possible pathway of psychosocial factors on risk of disease is via health behaviors [23]. Previous studies implicated the role of psychosocial factors in behavioral interventions. For example, in women with a history of gestational diabetes mellitus, an intervention aimed at psychosocial determinants to increase physical activity and better dietary behaviors showed improved confidence, support, and behavioral management skills [56]. This provides support for the development of interventions, which aim to change psychosocial determinants of lifestyle and to integrate these into methods for behavioral change. 

Our findings of limited associations between specific psychosocial factors and specific health risk behaviors, which are consistent with earlier studies (e.g., References [26,28,31,32]), do not suggest a need to tailor health promotion interventions regarding specific risk behaviors (e.g., smoking) to specific psychosocial factors or groups of factors (risks or resources). Although there are national guidelines for interventions on single behaviors, less support is available for interventions in the more complex situations of multiple health risk behaviors. As described above, engaging in multiple risk behaviors is related to a greater risk of disease than the summated risk for each of the behaviors [10,11,12,13] and, hence, there is a potential gain in multiple health risk behavior interventions. 

Our findings on prominent relationships of psychosocial factors to clustering of health risk behaviors emphasize the need for including psychosocial aspects in interventions targeting these multiple health risk behaviors. These psychosocial factors include social and psychological resources, as well as psychological risk factors. Our data suggest that trust, vital exhaustion, and depressiveness may be of special importance. It should be noted that these psychosocial factors comprise general resources and not behavior-specific constructs, such as attitude toward exercise, which are often included in socio-cognitive models (e.g., Reference [57]). One of the most known of these constructs is self-efficacy [22]. Perceived self-efficacy concerns people’s beliefs in their capabilities to control important factors in their lives which affect life choices and motivation to change. Self-efficacy is promoted by mastery experiences, successful role models, social persuasion of capabilities, and somatic and emotional states of personal strengths, quality of functioning, and resilience to stress and depression [22].Thus, self-efficacy, which is more situation-oriented, is related to general psychosocial factors of coping/mastery than to social support. A recent systematic review including longitudinal cohort studies on barriers and facilitators to healthy behaviors in mid-life populations (aged 40 to 64 years) illustrated the relevance of self-efficacy, but failed to find studies on other psychosocial factors [58]. Our results on psychosocial factors can offer valuable information on these factors; indeed, the findings suggest that, in addition to information and skills training, several psychosocial factors are important in behavior change, and these may act in concert [16]. 

Furthermore, self-esteem was the only factor that showed non-significant association with multiple health risk behaviors. In our previous studies, we demonstrated that self-esteem displayed independent associations to all three examined inflammatory markers (interleukin -6 (IL-6), C-reactive protein (CRP), and matrix metalloproteinase-9 (MMP-9)) after controlling for all four health risk behaviors. In parallel, the associations between hopelessness and IL-6 and CRP, and between depression and CRP and MMP-9 all disappeared after adjusting for health risk behaviors [59]. This suggests that the main effects of self-esteem on risk of coronary heart disease cannot solely be explained by an indirect pathway via health risk behaviors, but rather direct via effects on psychoneuroimmunological mechanisms [23].

One of the few studies investigating the relationship between self-esteem and health behaviors examined the number of cigarettes among smokers. Self-esteem showed a negative correlation with the number of cigarettes. In particular, an interaction effect was found where males with lower self-esteem exhibited more positive beliefs about smoking compared to women [32]. In future studies, possible interaction effects of psychosocial factors and behavior-specific factors would be interesting to explore, as well as gender aspects.

After controlling for the effects of all other psychosocial factors, trust was the only factor remaining statistically significant in the model. As trust was the instrument showing the smallest correlations to other factors, this could indicate an independent role of trust; however, this needs to be further investigated. Evidence supporting the effect of trust was found in a recent analysis of this data on 12-year coronary heart disease incidence where trust showed an independent effect (data to be published).

### 4.1. Methodological Considerations

A strength of the study is the unique design of the LSH research program, which allowed for comprehensive analyses of multiple health risk behaviors and psychosocial factors due to the broad range of variables. The well-characterized population-based sample also made it possible to adjust for known confounders. Although the statistical power of the study is a potential limitation, our findings consistently showed associations in the expected directions between health behaviors and psychosocial factors, with little indication that our conclusions or their generalizability would be affected by low power. After multiple-comparison correction carried out according to the false discovery rate (FDR), significance remained for eight out of nine factors. In these analyses, the risk of a type I error, associated with multiple testing, must be balanced toward the risk of a type II error, due to a too rigid analysis. We do suggest that, after the abovementioned control, our main message remains. Our correlation analysis showed, as expected, that all the psychosocial factors do correlate. These correlation coefficients are, in general, low to moderate, with the largest *r* being 0.6. The theoretical base for these instruments differs and, in earlier studies, we demonstrated that the choice of measures matters [60]. 

However, our aim was not to assess the different measures in relation to one another (examining, e.g., mediating or moderating factors), but instead to examine to what extent each of these, after control for confounding factors of life circumstances, is related to risk of having multiple health risk behaviors. Moreover, most often one or two psychosocial factors are and can be included in different studies, whether population-based or clinical. We, therefore, find it important to demonstrate the results for each of these. Finally, an important limitation is the cross-sectional design. We cannot draw conclusions on the directions of these relationships; we, therefore, do not know whether health risk behaviors are a result of or contribute to the psychosocial situation. Prospective studies are needed to assess the direction of associations. However, based on previous research, showing independent associations of psychosocial factors on the risk of disease [42], we do suggest that the main direction is the former, i.e., that psychosocial factors contribute to a capacity to change behavior. 

### 4.2. Implications for Practice

There is often a debate whether to focus on individual responsibility for behavior change or create structural conditions that promote and enable healthy lifestyles. It is obvious that structural factors are important and must be targeted, for example, increasing the capacity for support from the social environment to enhance positive outcome expectancies and health. This is also highlighted in health-oriented policies and the proposed “arena perspective” [61,62]. However, concurrently, empowerment strategies building on the ambition to enhance individual chances of developing positive expectancies, hopes, and trust are needed [63]. Our findings illustrate the importance of identifying vulnerable individuals with loss of coping in terms of vital exhaustion and depressiveness, especially in the case of multiple health risk behaviors.

## 5. Conclusions

Examining associations between a broad range of psychosocial factors and multiple health risk behaviors revealed consistent and significant associations for almost all psychosocial factors. These associations were more prominent compared to analyzing associations to single health risk behaviors. Our findings support the relevance of considering psychosocial aspects in interventions aimed at health behavior change, especially for people with multiple health risk behaviors.

## Figures and Tables

**Table 1:** Demographic characteristics, health behaviors, and socioeconomic status of the study population.

Factors and Status	Variable	Category	*n* (%)	Mean (SD) or Median (Q1; Q3)
Demographics	Age; mean (SD)		1007 (100)	57 (7.2)
	Sex	Female	505 (50)	
		Male	502 (50)	
Health behavior	Smoking	Non-smoker	785 (81)	
		Current smoker	183 (19)	
				
	Alcohol intake	Below risk level	869 (86)	
		Risk level *	138 (14)	
				
	Physical activity	Sufficient **	176 (19)	
		Insufficient	757 (81)	
				
	Fruit and vegetable intake	Sufficient ***	195 (20)	
		Insufficient	795 (80)	
				
	Number of unhealthy behaviors	0	47 (5)	
		1	214 (24)	
		2	453 (50)	
		3	162 (18)	
		4	30 (3)	
Socioeconomic	Education (school years)	≤9 years (compulsory school)	352 (36)	
Status		10–11 years	294 (30)	
		12–13 years	133 (14)	
		≥14 years (university)	209 (21)	
				
	Immigrant status	Born in the Nordic countries	945 (95)	
		Born outside the Nordic countries	54 (5)	
				
	Employment information	Employed	614 (61)	
		Unemployed	111 (11)	
		Retired	282 (28)	

* Risk level is drinking more than nine standard glasses per week for women and more than 14 glasses per week for men, and/or reporting drinking four or more standard glasses for women and five or more glasses for men on a typical day when drinking; ** sufficient is the highest category out of four, based on a combination of regular physical activity during daytime and physical training; *** sufficient is an intake of ≥500 g of fruit and vegetables per day.

**Table 2:** Prevalence of health behaviors and their combinations.

Health Behavior	*n*	Percentage
Insufficient physical activity + insufficient intake of fruit and vegetables	416	45.9
Insufficient intake of fruit and vegetables	151	16.7
Insufficient physical activity	105	11.6
Current smoker + insufficient physical activity + insufficient intake of fruit and vegetables	91	10
Risky alcohol intake + insufficient physical activity + insufficient intake of fruit and vegetables	70	7.7
Current smoker + risky alcohol intake + insufficient physical activity + insufficient intake of fruit and vegetables	30	3.3
Risky alcohol intake + insufficient intake of fruit and vegetables	12	1.3
Current smoker + insufficient physical activity	11	1.2
Risky alcohol intake + insufficient physical activity	10	1.1
Current smoker + insufficient intake of fruit and vegetables	4	0.4
Risky alcohol intake	4	0.4
Current smoker	1	0.1
Current smoker + risky alcohol intake + insufficient intake of fruit and vegetables	1	0.1
Total	906	100

**Table 3:** Characteristics of psychosocial factors in the study population.

Psychosocial Scales ^1^	Number of Items	Cronbach’s Alpha	Range in Scale	Range in Study Population	Mean (SD)	Median (Q1; Q3)	Number of Responders
Psychosocial resources							
Social integration	6	0.88	6–36	6–36	20.5 (5.9)	20 (16; 24)	962
Emotional support	6	0.77	0–6	0–6	5.5 (1.1)	6 (5; 6)	964
Perceived control	11	0.69	0–55	15–55	39.8 (7.7)	40 (35; 46)	894
Self-esteem	10	0.86	10–40	15–40	32.2 (4.8)	33 (30; 36)	947
SOC ^2^	13	0.82	13–91	32–91	68.7 (10.4)	70 (62; 77)	962
Trust	1	-	1–5	1–5	4.0 (0.6)	4 (4; 4)	959
Psychological risk factors							
Cynicism	12	0.85	12–60	12–53	31.2 (7.71)	32 (26; 37)	969
Vital exhaustion	19	0.94	19–57	19–56	30.2 (7.63)	29 (24; 35)	965
Hopelessness	2	0.70	0–8	0–8	2.28 (2.03)	2 (1; 4)	963
Depressiveness	20	0.68	0–60	0–51	9.02 (7.86)	7 (3; 12)	937

^1^ A high score on the psychosocial resource scales is considered desirable, while a high score on a psychosocial risk scale is considered non-desirable. ^2^ Sense of coherence.

**Table 4:** Correlation matrix for psychosocial factors.

	Social Integration	Emotional Support	Perceived Control	Self Esteem	SOC	Trust	Cynicism	Vital Exhaustion	Hopelessness	Depressiveness
Psychosocial resources										
Social integration	1	0.377	0.309	0.363	0.360	0.206	−0.194	−0.283	−0.296	−0.337
Emotional support		1	0.188	0.239	0.234	0.134	−0.159	−0.197	−0.231	−0.256
Perceived control			1	0.466	0.428	0.187	−0.313	−0.422	−0.517	−0.488
Self esteem				1	0.571	0.145	−0.190	−0.563	−0.503	−0.515
SOC ^1^					1	0.264	−0.319	−0.600	−0.428	−0.528
Trust						1	−0.308	−0.151	−0.186	−0.148
Psychological risk factors										
Cynicism							1	0.107	0.276	0.150
Vital exhaustion								1	0.423	0.671
Hopelessness									1	0.447
Depressiveness										1

^1^ Sense of coherence.

**Table 5:** Associations between health risk behaviors and psychosocial factors. Odds ratios (ORs) and confidence intervals (CIs) according to logistic regression for health risk behavior, adjusted (adj) for age, sex, education, employment, and immigrant status, *n* ranging from 810 to 933.

	Current Smoker			Risky Alcohol Intake			Insufficient Physical Activity			Insufficient Intake of Fruit and Vegetables		
	Adj OR	95% CI	*p*	Adj OR	95% CI	*p*	Adj OR	95% CI	*p*	Adj OR	95% CI	*p*
Psychosocial resources												
Social integration	0.94	0.79–1.13	0.519	0.88	0.72–1.07	0.195	0.90	0.75–1.08	0.243	0.86	0.72–1.02	0.076
Emotional support	0.84	0.72–0.97	0.018	0.76	0.65–0.90	0.001	0.96	0.79–1.15	0.641	0.87	0.72–1.05	0.156
Perceived control	0.79	0.66–0.95	0.014	0.84	0.67–1.04	0.104	0.73	0.59–0.89	0.002	1.02	0.86–1.23	0.777
Self esteem	0.90	0.76–1.07	0.252	0.86	0.71–1.05	0.143	0.97	0.82–1.16	0.762	0.90	0.75–1.07	0.214
SOC ^1^	0.84	0.71–1.00	0.055	0.79	0.64–0.96	0.019 *	0.79	0.65–0.95	0.013	0.98	0.82–1.16	0.780
Trust	0.84	0.71–0.99	0.034 *	0.73	0.61–0.88	0.001	0.85	0.70–1.03	0.093	0.92	0.77–1.09	0.330
Psychological risk factors												
Cynicism	1.18	0.99–1.41	0.063	1.19	0.97–1.46	0.101	1.21	1.01–1.44	0.040 *	1.02	0.86–1.21	0.837
Vital exhaustion	1.36	1.14–1.61	0.001	1.25	1.02–1.54	0.033 *	1.34	1.11–1.63	0.003	0.99	0.84–1.18	0.942
Hopelessness	1.14	0.96–1.36	0.143	1.07	0.87–1.32	0.511	1.29	1.05–1.58	0.013	1.10	0.92–1.32	0.311
Depressiveness	1.51	1.28–1.79	<0.001	1.20	0.98–1.47	0.082	1.23	1.01–1.50	0.036 *	1.11	0.93–1.33	0.245

* *p* > 0.05 after correction for multiple testing (false discovery rate (FDR)). ^1^ Sense of coherence.

**Table 6:** Relationships between psychosocial factors and number of health risk behaviors (0–1, 2, or 3–4). Odds ratios according to ordinal logistic regression for falling in a higher level of health risk behaviors, adjusted for age, sex, education, employment status, and immigrant status, *n* ranging from 813 to 874.

	Adj OR	95% CI	*p*
Psychosocial resources			
Social integration	0.88	0.77–0.99	0.047 *
Emotional support	0.81	0.70–0.92	0.002
Perceived control	0.84	0.73–0.97	0.020
Self esteem	0.93	0.81–1.06	0.270
SOC ^1^	0.84	0.73–0.95	0.008
Trust	0.80	0.70–0.91	0.001
Psychological risk factors			
Cynicism	1.18	1.03–1.35	0.014
Vital exhaustion	1.28	1.11–1.46	<0.001
Hopelessness	1.19	1.03–1.37	0.016
Depressiveness	1.32	1.14–1.52	<0.001

* *p* > 0.05 after correction for multiple testing (FDR). ^1^ Sense of coherence.
